# Radon and risk of extrapulmonary cancers: results of the German uranium miners' cohort study, 1960–2003

**DOI:** 10.1038/sj.bjc.6604776

**Published:** 2008-11-11

**Authors:** M Kreuzer, L Walsh, M Schnelzer, A Tschense, B Grosche

**Affiliations:** 1 Federal Office for Radiation Protection, Department of Radiation Protection and Health, Neuherberg 85764, Germany

**Keywords:** epidemiology, radon, radiation, cohort study, U-miners

## Abstract

Data from the German miners' cohort study were analysed to investigate whether radon in ambient air causes cancers other than lung cancer. The cohort includes 58 987 men who were employed for at least 6 months from 1946 to 1989 at the former Wismut uranium mining company in Eastern Germany. A total of 20 684 deaths were observed in the follow-up period from 1960 to 2003. The death rates for 24 individual cancer sites were compared with the age and calendar year-specific national death rates. Internal Poisson regression was used to estimate the excess relative risk (ERR) per unit of cumulative exposure to radon in working level months (WLM). The number of deaths observed (O) for extrapulmonary cancers combined was close to that expected (E) from national rates (*n*=3340, O/E=1.02; 95% confidence interval (CI): 0.98–1.05). Statistically significant increases in mortality were recorded for cancers of the stomach (O/E=1.15; 95% CI: 1.06–1.25) and liver (O/E=1.26; 95% CI: 1.07–1.48), whereas significant decreases were found for cancers of the tongue, mouth, salivary gland and pharynx combined (O/E=0.80; 95% CI: 0.65–0.97) and those of the bladder (O/E=0.82; 95% CI: 0.70–0.95). A statistically significant relationship with cumulative radon exposure was observed for all extrapulmonary cancers (ERR/WLM=0.014%; 95% CI: 0.006–0.023%). Most sites showed positive exposure–response relationships, but these were insignificant or became insignificant after adjustment for potential confounders such as arsenic or dust exposure. The present data provide some evidence of increased risk of extrapulmonary cancers associated with radon, but chance and confounding cannot be ruled out.

Although it is well established that occupational exposure to the radioactive gas, radon (^222^Rn), and its progeny increases the risk of lung cancer (BEIR, 1999; Laurier *et al*, 2004; Tomasek, 2004; Brüske-Hohlfeld *et al*, 2006; Grosche *et al*, 2006; Villeneuve *et al*, 2007; Tomasek *et al*, 2008; Vacquier *et al*, 2008), little is known about any effects on other cancers (Tomasek *et al*, 1993; Darby *et al*, 1995a; BEIR, 1999; Moehner *et al*, 2006; Rericha *et al*, 2006). As it is estimated that doses from radon and its progeny to organs other than the lung are approximately ⩾100 times lower (Kendall and Smith, 2002; Marsh *et al*, 2008), large-scale occupational radon studies are required to investigate this possible relationship.

The largest and most informative study on this subject to date is the pooled analysis of 11 miners’ cohorts published by Darby *et al* (1995a). Overall, no statistically significant exposure–response relationship was observed, except for pancreatic cancer. The researchers concluded that high concentrations of radon in the air do not cause a material risk of mortality from cancers other than lung cancer. However, low statistical power, missing information on potential confounders and heterogeneity among the 11 studies were of concern. The aim of these analyses of the German Wismut uranium miners’ cohort study is to further evaluate the relationship between radon and extrapulmonary cancers. Compared with the pooled study, the Wismut cohort has a comparable size (58 987 *vs* 64 209), a longer average follow-up period (35 *vs* 17 years), a larger number of deaths from cancers other than lung cancer (3340 *vs* 1253), a longer mean duration of employment (12 *vs* 6 years) as well as a higher average cumulative exposure to radon (279 *vs* 155 working level months (WLM)). Moreover, information on occupational exposure to external *γ*-radiation, long-lived radionuclides (LRNs), arsenic, fine dust and silica dust is available.

## Materials and methods

### Cohort definition and follow-up

The cohort has been described earlier (Grosche *et al*, 2006; Kreuzer *et al*, 2002, 2006). In brief, it represents a stratified random sample of 58 987 males, employed for at least 6 months from 1946 to 1989 at the former Wismut uranium company in East Germany. The first follow-up ran up to the end of 1998 (Grosche *et al*, 2006; Kreuzer *et al*, 2006), and this study extends the follow-up by 5 years through 2003. Information on the vital status of individuals was obtained from local registration offices, whereas death certificates were obtained not only from the responsible Public Health Administrations but also from central archives and the pathology archive of the Wismut company. The underlying causes of death from death certificates or the autopsy files were coded according to the 10th revision of the International Classification of Diseases (ICD-10).

### Information on exposure to radiation and other variables

Radiation exposure was estimated by using a job-exposure matrix (JEM), which was originally generated for compensation purposes by the Miners’ Institution for Statutory Accident Insurance and Prevention (Lehmann *et al*, 1998). This JEM has been developed further to meet scientific purposes (HVBG and BBG, 2005). It includes information on exposure to radon and its progeny, external *γ*-radiation and LRNs (^235^U and ^238^U) for each calendar year of employment (1946–1989), each place of work and each type of job. More than 900 different jobs and 500 different working places were evaluated for this purpose. Radon (^222^Rn) measurements in the Wismut mines were carried out from 1955 onwards. For the period 1946–1954, radon concentrations were estimated by an expert group based on measurements from 1955, taking into account ventilation rate, vein space, uranium content and so on (Grosche *et al*, 2006; Kreuzer *et al*, 2006; Moehner *et al*, 2006). Complete information on job type, type of mining facility and periods of absence is available on a daily basis for all cohort members. The cumulative exposure to each of the three radiation sources was calculated as the sum of the annual exposures estimated by the JEM weighted by the duration of work in days. Exposure to radon and its progeny is expressed in WLM. One working level is defined as the concentration of short-lived radon daughters per litre of air that gives rise to 1.3 × 10^5^ MeV of alpha energy after complete decay. One WLM of cumulative exposure corresponds to exposure to 1 WL during 1 month (170 h) and is equivalent to 3.5 mJh m^−3^. Exposure to LRNs is given in kBqh m^−3^, and exposure to external *γ*-radiation is given as an effective dose in mSv.

Information on arsenic, dust and silica is based on a JEM similar to that for radiation, providing annual dose values for each calendar year, each place of work and each type of job (Bauer, 2000; HVBG and BBG, 2005). These annual values are given in dust-years, where 1 dust-year is defined as an exposure to 1 mg m^−3^ fine dust or silica dust and 1 *μ*g m^−3^ for arsenic over a time period of 220 shifts of 8 h. Differences in the number of shifts and daily working hours in the different calendar years were accounted for by multiplying with a correction factor. Cumulative exposure to each of the three sources is expressed in dust-years. Arsenic was present only in mines in Saxony. Thus, arsenic exposure was calculated only for cohort members who worked in areas with rock containing a sufficient concentration of arsenic. The threshold value for arsenic is 10 *μ*g m^−3^ in air (inhaled particle fraction), corresponding to one-tenth of the technical guideline concentration value of arsenic, which was valid until 2004. Data on smoking are not included in this analysis because the relevant information is only available after 1972 from medical records for a small proportion of the cohort.

### Statistical methods

Two statistical methods were applied, external comparisons with national rates and internal regression. In the first method, the mortality rates of the cohort were compared with those of the general male population in Eastern Germany, formerly the German Democratic Republic. External rates were available only from 1960 onwards. For this reason, all analyses were limited to the follow-up period 1960–2003, with the 236 cohort deaths before 1960 being excluded. The number of man-years at risk for each miner was calculated as the time between entry into and exit from the cohort. In these analyses, the date of entry was defined as the start of employment plus 180 days or 1 January 1960, whichever comes later. The date of exit was defined as the earliest of the date of death, emigration, loss to follow-up or the end of the period of follow-up (31 December 2003). The expected mortality rate was calculated by applying national mortality rates, grouped by calendar year and 5-year age bins, to the person-years in the grouped cohort data. The standardised mortality ratio (SMR) is given by the ratio of O/E, where O is the number of observed deaths in the cohort and E is the number expected from external rates. In common with other miners’ studies (Tomasek *et al*, 1993; Darby *et al*, 1995a), a 5-year lag was used to calculate the cumulative exposure to radon for all sites of cancer other than leukaemia and a zero lag for leukaemia. The confidence intervals of the SMR were calculated on the basis of the poisson distribution (Breslow and Day, 1987). SMRs were corrected for missing causes of death by dividing O by the proportion of known causes of death, *P*, which is binomial distributed. In practice, it was found to be adequate to ignore the variability of *P* as, when methods were applied to account for this variability (Rittgen and Becker, 2000), the resulting SMR confidence intervals were not significantly affected.

The cancer sites examined were defined according to the pooled study by Darby *et al* (1995a), but the 10th ICD code was used instead of the 9th ICD code. Earlier revisions of the ICD (8 and 9) and the former codes of the German Democratic Republic were recoded to ICD-10. As no separate external rates had been available for the time period 1968–1979 for cancers of the tongue and mouth, salivary gland and pharynx, these cancers were combined in the external analyses. In a few cases, the external rates were not available for certain years and cancer types and hence were not included in the corresponding external analyses, which is why the total numbers are sometimes lower than those in the internal regression and do not sum to the total number of non-lung cancers. A separate analysis for time periods < or >10 years since first employment was performed, because earlier studies (Tomasek *et al*, 1993; Darby *et al*, 1995a) showed a lower mortality during the first period compared with the later period, most probably because of the selection of healthy men for employment in the mines. Owing to the long follow-up period in this cohort and the restriction of the follow-up period to 1960 and later, the proportion of cases occurring <10 years after the first employment was extremely low (1.7%) and thus did not affect the overall risk estimate.

Poisson regression was used to test for an association between cancer mortality risk and cumulative radon exposure. Tabulations of person-years at risk and cancer deaths were created with the DATAB module of the EPICURE software (Preston *et al*, 1998). Cross-classifications were made by age, *a*, in 16 categories (<15, 15–19, 20–24,…, 85+ years), individual calendar year, *y*, in 58 categories and cumulative radon exposure, *w*, in seven categories (0, >0–49, 50–99, 100–499, 500–999, 1,000–1,499, 1500+ WLM). The WLM categories were defined to be comparable with other studies (Darby *et al*, 1995a; Moehner *et al*, 2006), but with an added category of 0 WLM. The tabulated data were fitted to the following model – if *r*(*a, y, w*) is the age, year and exposure-specific cancer mortality rate and *r*_*0*_(*a,y*)=*r*(*a,y,0*) is the baseline disease rate for non-exposed individuals, *w*=0 then 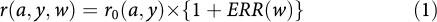 where ERR is the excess relative risk. A linear form for ERR(*w*) =*βw*, with no dependence of the slope *β* on *a* and *y*, was used to investigate the exposure–response relationship. In addition, a categorical analysis of the form ERR(*w*)=Σ_*j*=1,7_*β*_*j*_*w*_*j*_ was performed, where *j* refers to the exposure class. To test for the five potential confounders, LRN, external *γ*-radiation, fine dust, quartz fine dust or arsenic, each of these variables (*z*_*i*_), *i*=1, 5 was added separately to the model (1) with ERR(*w*, *z*_*i*_)=*βw*+*γz*_*i*_. Maximum likelihood with the AMFIT module of the EPICURE software (Preston *et al*, 1998) was used for estimation of the fit parameters: *β*, *γ*, *β*_*j*_ (*j*=1, 7), and the internal baseline rates in strata. Internal regression analyses were restricted to individual cancer sites with a total of >35 deaths.

## Results

In the follow-up period 1960–2003, a total of 57 199 persons were under observation, resulting in 1 762 208 person-years at risk and a mean duration of follow-up of 35 years. By the end of 2003, 35 294 (61.7%) men were alive, 20 684 (36.2%) had died, 233 (0.4%) had emigrated and 988 (1.7%) were lost to follow-up. The underlying cause of death was available for 19 501 (94.3%) of the deceased men, among them 6341 deaths from malignant cancers (2999 lung cancers plus two cancers of the trachea and 3340 non-lung cancers). A total of 49 268 individuals were exposed to radon at some time during Wismut employment, whereas 7931 had never been exposed (Table 1). Those exposed received a mean cumulative exposure to radon of 279.4 WLM (median=30.8), a mean cumulative exposure to external *γ*-radiation of 48.6 mSv (median=16.5) and an average cumulative exposure to LRN of 4.2 kBqh m^−3^ (median=1.05).

**Figure 1:**
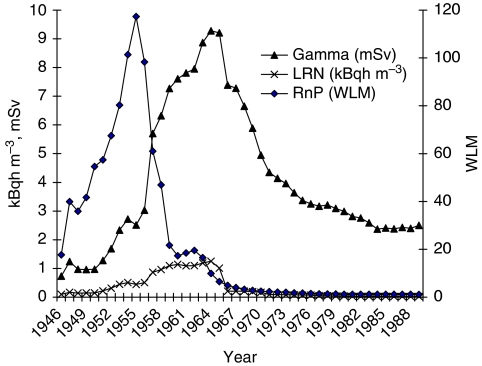
Mean annual exposure to radon and its progeny in working level months (WLM), external *γ*-radiation in mSv and long-lived radionuclides in kBqh m^−3^ among exposed miners.

**Figure 2:**
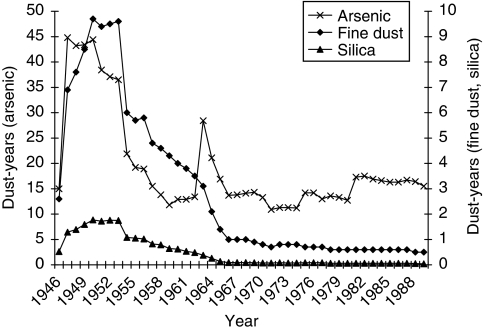
Mean annual cumulative exposure for exposed cohort members with respect to fine dust (*n*=56 914), silica dust (*n*=56 878) and arsenic exposure (*n*=17 554) in dust-years.

Figure 1 shows the annual mean exposure values for radon and its progeny in WLM, and for external *γ*-radiation in mSv and LRN in kBqh m^−3^ for the exposed cohort members. Radon concentrations decreased sharply after 1955 because of the introduction of several ventilation measures, which led to conditions in accordance with the international radiation protection standards after 1970. In contrast to this, external *γ*-radiation and LRN show a different pattern, because their concentration was not affected by the improved ventilation. The annual mean exposure values for fine dust, silica dust and arsenic are given in Figure 2. Owing to the use of dry drilling, the concentrations of dust had been very high until 1955 and then decreased steadily with the implementation of wet drilling, reaching very low levels after 1970. A total of 17 554 miners were exposed to arsenic, with higher annual values in the early years compared with the later years.

Table 2 gives the numbers of O and E deaths based on the male Eastern German population, as well as the corresponding SMRs (O/E) with 95% CIs for all cancers other than lung cancer combined and for 24 individual cancer sites. The number of non-lung cancer deaths combined was close to expectation (O/E=1.02; 95% CI: 0.98–1.05). Among 24 individual non-lung cancer sites, a significant excess was found for stomach (O/E=1.15; 95% CI: 1.06–1.25) and liver cancers (O/E=1.26; 95% CI: 1.07–1.48), as well as a significant deficit of cancers of the tongue, mouth, pharynx and salivary gland combined (O/E=0.80; 95% CI: 0.65–0.97) and those of the bladder (O/E=0.82; 95% CI: 0.70–0.95). Overall mortality was significantly higher than in the general population (O/E=1.03; 95% CI: 1.02–1.05), mainly because of lung cancer (O/E=2.03; 95% CI: 1.96–2.10).

In the internal regression analyses shown in Table 3, there is a significantly increased mortality from all cancers other than lung cancer with cumulative radon exposure (ERR/WLM=0.014%; 95% CI: 0.006–0.023%). The two highest exposure categories 1000–1499 WLM and >1500 WLM show a 1.2-fold (95% CI: 1.02–1.38) and 1.16-fold (95% CI: 0.94–1.76) higher risk compared with the reference category of 0 WLM, respectively. Among the 18 individual sites with >35 cases, a significant positive relation with radon is observed for stomach cancer (ERR/WLM=0.021%; 95% CI: 0.0007–0.043%), whereas excesses with borderline statistical significance were found for cancers of the pharynx (ERR/WLM=0.16%; 95% CI: −0.045 to 0.37%) and liver (ERR/WLM=0.044%; 95% CI: −0.008 to 0.096%). No association between leukaemia and cumulative radon exposure is found. This is also true for all leukaemia except chronic lymphatic leukaemia (non-CLL) (*n*=87, ERR/WLM=0.019%; 95% CI: −0.04 to 0.08%), CLL (*n*=40, ERR/WLM=−0.013%; 95% CI: −0.067 to 0.040%) and acute myeloid leukaemia (*n*=31, ERR/WLM=0.036%; 95% CI: −0.076 to 0.149%).

Overall, there is a low correlation between exposure to radon and exposure to external *γ*-radiation, LRN or arsenic (*R*<0.28), whereas fine dust (*R*=0.57) and silica dust (*R*=0.63) are relatively highly correlated with radon exposure. Additional adjustment for each of the five factors showed no substantial modifying effect on the overall ERR/WLM for all non-lung cancers combined. In contrast, the adjustment led to a decreased risk for certain sites (e.g., stomach, larynx and liver) (Table 4). Overall, none of the risk estimates for the different cancer sites were significant after adjustment for the potential confounders.

## Discussion

In this study, a statistically significant relation between cumulative radon exposure and risk of extrapulmonary cancers combined is observed (ERR/WLM=0.014%). After adjustment for potential confounders, such as exposure to arsenic, dust, LRN and *γ*-radiation, the ERR/WLM is only marginally modified, values of the ERR/WLM ranging from 0.016 to 0.011%, with some of the borderline significance. No earlier miners’ studies have reported a statistically significant result for this relationship (Tomasek *et al*, 1993; Darby *et al*, 1995a; Vacquier *et al*, 2008), and hence a non-causal chance result in our study cannot be ruled out. However, the earlier studies may have been limited by low statistical power. For example, in the pooled study by Darby *et al* (1995a) an ERR/WLM of 0.01% for the time period >10 years after employment was observed, in line with our findings, but it is not statistically significant (Table 5). In both studies, an excess of non-lung cancers seems to be present only for exposure categories above 1000 WLM.

**Figure 3:**
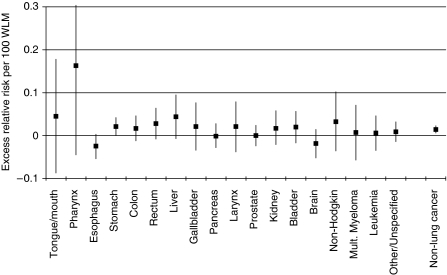
Excess relative risk (ERR) per 100 WLM and 95% confidence limits for all cancer sites with >35 cases and all cancers other than lung cancer combined, 1960–2003.

Dosimetric calculations indicate that extrapulmonary organs received very low doses compared with those received by the lung (Kendall and Smith (2002); Marsh *et al*, 2008). Marsh *et al* (2008) recently estimated the absorbed doses for specific organs for several exposure scenarios in mines. For example, wet drilling, medium ventilation and medium physical activities were associated with the following doses in mGy/WLM: bronchial region 7.3, red bone marrow 0.031, kidney 0.02 and liver 0.0065. In our analyses, the ERR/WLM for lung cancer is approximately 14 times higher (*n*=2999; ERR/WLM=0.20%; 95% CI: 0.16–0.22%) than for non-lung cancers (*n*=3,340; ERR/WLM=0.014%), which is compatible with the biokinetic models. For individual sites, the majority showed a positive exposure–response relationship (15 from 18), although this was significant only for stomach cancer (Figure 3). After adjusting for the five potential confounders, however, no individual sites showed a significant exposure–response relationship.

### Specific sites

#### Liver

The increased mortality of liver cancer in miners compared with the general population (*n*=158, O/E=1.26; 95% CI: 1.06–1.25) is consistent with other miners’ studies (Tomasek *et al*, 1993; Darby *et al*, 1995a, 1995b) and appears not to be a chance finding. It may be because of the high consumption of alcohol among miners, which, in the early years, was offered (with cigarettes) free of charge. Alcohol abuse, or cirrhosis, was mentioned on the death certificate for 8%, or 37%, of the liver cancers, respectively. The principal two studies provided no evidence of a relationship with increasing cumulative exposure to radon (Tomasek *et al*, 1993; Darby *et al*, 1995a). In contrast, a non-significantly elevated ERR/WLM of 0.044% (*P*=0.09) is observed here. Adjustment for exposure to external *γ*-radiation, LRN, arsenic and dust led to some decrease in the ERR/WLM. Confounding from other factors such as alcohol consumption cannot be ruled out.

#### Stomach

A significantly elevated SMR for stomach cancer (*n*=590, O/E=1.15; 95% CI: 1.06–1.25) was observed here, as in other studies of radon-exposed miners (Kusiak *et al*, 1993; Darby *et al*, 1995a), and among coal miners (Rockette, 1977). Although not fully understood, it could be related to dust exposure (Cocco *et al*, 1996). In the pooled study, an elevated SMR (*n*=217; SMR=1.33; 95% CI: 1.16–1.52) was found with no exposure–response relationship (Darby *et al*, 1995a), whereas in our study, the risk increased significantly with increasing cumulative radon exposure (ERR/WLM=0.022%). The highest exposure category (1500 WLM or more) was associated with a 1.8-fold (95% CI: 1.06–2.48) significantly higher risk of death compared with the reference category of 0 WLM. Adjustment for each of the five confounders, however, reduced the ERR/WLM by a factor of approximately 2, leading to insignificant values. Thus, part of the proportionate increase in risk because of radon might be explained by confounding.

#### Pharynx

A significant deficit of cancers of the tongue, mouth, salivary gland and pharynx combined (*n*=99, SMR=0.8; 95% CI: 0.65–0.97) may be a chance finding because of multiple testing. There was a constant, but not significant, increase in pharyngeal cancer risk with increasing cumulative radon exposure (*n*=53, ERR/WLM=0.16%; 95% CI: −0.045 to 0.37%). It can be noted that this value was nearly as high as for lung cancer (ERR/WLM=0.20%), but no such relationship was reported in other studies on miners, although the number of pharyngeal cases was small (Tomasek *et al*, 1993; Darby *et al*, 1995a). Additional adjustment for the five possible confounders only led to a small reduction of the ERR/WLM. Some studies have provided estimates for organ doses after inhalation of radon and its progeny separately for the extrathoracic airways, and have reported a pharyngeal dose that was nearly as high as the lung dose (Kendall and Smith 2002; Jacobi and Roth, 1995).

#### Larynx

The combined 11 studies on miners showed a 1.21-fold non-significantly increased SMR for larynx cancer that was not related to cumulative radon exposure (Darby *et al*, 1995a), but there were only 38 cases. In the first follow-up of the French uranium miners’ study (1946–1985), a significantly increased SMR of 2.35 was observed on the basis of 17 cases (Tirmarche *et al*, 1993), which became insignificant after extension of the follow-up period to 1999 (SMR=1.24, *n*=29) (Vacquier *et al*, 2008). The SMR in this study (*n*=75, SMR=1.18; 95% CI: 0.93–1.48) is comparable with the findings of the pooled study (Darby *et al*, 1995a). ERR/WLM was elevated, but not significantly. Adjustment for the five potential confounders led to a substantial decrease in the observed ERR/WLM.

#### Kidney

Animal experiments suggest an increased mortality of kidney cancer after inhalation of radon (Masse *et al*, 1992), but none of the miners’ studies found any such excess (Tomasek *et al*, 1993; Darby *et al*, 1995a), apart from the French study (*n*=20, SMR=2.0; 95% CI: 1.22–3.09) (Vacquier *et al*, 2008). Moreover, none of these studies observed a trend with cumulative radon exposure. The same holds true in our data, there being no excess (*n*=162, SMR=0.91) or an exposure–response relationship.

#### Leukaemia

In our study, no association between cumulative radon exposure and leukaemia is found, or with CLL, non-CLL or AML, consistent with earlier studies (Tomasek *et al*, 1993; Darby *et al*, 1995a; Laurier *et al*, 2001; Vacquier *et al*, 2008), including a recent large case–control study with 377 leukaemia cases among former Wismut employees (Moehner *et al*, 2006). In contrast, Rericha *et al* (2006) noted, in a Czech uranium miner case–cohort study, a significantly increased relative risk of 1.75 (95% CI: 1.10–1.75) for leukaemia incidence in the highest quintile of cumulative radon exposure (>100 WLM) compared with the lowest (<3 WLM). However, there was a very high correlation in the mines, between radon and exposure to *γ*-radiation, which could have introduced confounding bias.

### Strengths and limitations

The major strengths of our study are the large cohort size, the large number of extrapulmonary cancers, the long follow-up period, the wide range of radon exposures and particularly the information available on other exposures such as arsenic, fine dust, silica, external *γ*-radiation and LRN. These advantages allowed the independent replication of the analysis of the 11 miners’ cohort studies (Darby *et al*, 1995a), which may have suffered from heterogeneity problems. The potential limitations of this study include the accuracy of the underlying causes of death on death certificates, missing causes of death, exposure misclassification particularly in the early years of mining activities as well as missing information on other potential confounders such as alcohol consumption, smoking, occupational exposure to diesel exhaust or asbestos. Moreover, despite the large number of cancer cases overall, there is a low statistical power with respect to certain sites, and multiple testing could have led to some spurious findings.

### Confounding

Within a nested case–control study of lung cancer in the Wismut cohort, information on smoking was collected from miners, their relatives and the medical Wismut archive. Most of the former Wismut employees had been smokers. Overall, the low correlation between smoking and cumulative radon exposure makes smoking an unlikely major confounder. It is known that Wismut employees in the early years had a relatively high alcohol consumption compared with the male general population. For approximately 5% of the deceased cohort members, alcohol abuse was noted on the death certificate. This rough surrogate for alcohol consumption was slightly negatively correlated with cumulative radon exposure.

### Exposure misclassification

Inevitably, exposures in the very early years are associated with considerable uncertainty. To obtain some insight into potential bias by misclassification, the cumulated radon exposure was separated into two components according to other studies investigating the effect of the quality of exposure (Tomasek *et al*, 2008; Vacquier *et al*, 2008), one risk estimate for the period 1946–1954, the years with retrospectively estimated radon concentrations and the other for when the JEM was based on measurements in the shafts. As, for all non-lung cancers combined, there was only a non-significant difference in the estimates for these two periods, a major bias through misclassification of exposure is unlikely, but cannot be excluded. Another potential limitation is the use of the exposure to radiation instead of the actual organ dose. Recently, it has been suggested that several factors such as physical activity, ventilation in the mines, dry or wet drilling may influence the individual doses (Marsh *et al*, 2008). Work on these dose calculations is currently in progress within the European collaborative research project ALPHA-RISK (European Commission, 2006), which will also provide a method for calculating the dose to the various organs from combined exposure to radon and its progeny, LRN and external *γ*-radiation.

## Conclusion

Some evidence of a very small radon-related risk of extrapulmonary cancers was found, compatible with dosimetric calculations for organ doses. However, the possibility of non-causal results because of chance and confounding cannot be ruled out.

## Figures and Tables

**Table 1:** Characteristics of the German uranium miners' cohort, 1960–2003

	**Mean (minimum–maximum)**
Duration of follow-up (years)	35 (0.5–50)
Duration of employment (years)	12 (0.5–40)
	
*Radon exposed miners (n*=*49.268)*	
Duration of exposure (years)	11 (1–44)
Age at first exposure (years)	22 (14–67)
Cumulative exposure (WLM^a^)	279 (>0–3224)
	
*Miners exposed to*	
External *γ*-radiation (*n*=49 256)	
Cumulative exposure (mSv)	48.6 (>0–908.6)
Long-lived radionuclides (*n*=49 256)	
Cumulative exposure (kBqh m^−3^)	4.2 (>0–132.2)
Arsenic (*n*=17 554)	
Cumulative exposure (dust-years^b^)	122.5 (>0–1417.4)
Fine dust (*n*=56 914)	
Cumulative exposure (dust-years^c^)	36.7 (>0–315.2)
Silica dust (*n*=56 878)	
Cumulative exposure in (dust-years^c^)	5.8 (>0–56.0)

a WLM=working level months.

b Dust-year is defined as exposure to 1 *μ*g m^−3^ for arsenic over 220 shifts each at 8 h.

c Dust-year=1 dust-year is defined as exposure to 1 mg m^−3^ of fine dust or silica dust over 220 shifts each at 8 h.

**Table 2:** Number of deaths observed (O) and expected (E), ratio of observed to expected deaths (O/E), and 95% CI for selected cancer sites, 1960–2003

**Cancer site (ICD-10 code)**	**O**	O*	**E**	O*/E	95% CI*
Tongue, mouth, salivary gland and pharynx (C00–C14)	99	105.0	131.8	0.80	0.65–0.97
Oesophagus (C15)	125	132.6	120.1	1.10	0.92–1.31
Stomach (C16)^a^	588	623.7	542.2	1.15	1.06–1.25
Colon (C17–C18)	299	317.1	310.9	1.02	0.91–1.14
Rectum (C19–C21)	241	255.6	267.9	0.95	0.84–1.08
Liver (C22)^a^	154	163.3	129.3	1.26	1.07–1.48
Gallbladder (C23–C24)^a^	76	80.6	75.4	1.07	0.84–1.34
Pancreas (C25)^a^	223	236.5	225.2	1.05	0.92–1.20
Nose (C30–C31)^a^	8	8.5	8.0	1.06	0.46–2.09
Larynx (C32)	75	79.5	67.2	1.18	0.93–1.48
Bone (C40–C41)	13	13.8	21.4	0.64	0.34–1.10
Malignant melanoma (C43)^a^	33	35.0	48.0	0.73	0.50–1.02
Other skin (C44)	9	9.5	12.5	0.76	0.35–1.45
Connective tissue (C47 and C49)^a^	14	14.8	21.6	0.69	0.38–1.16
Prostate (C61)^a^	262	277.9	314.4	0.88	0.78–1.00
Testis (C62)	25	26.5	25.3	1.05	0.68–1.55
Kidney (C64–C66)^a^	162	171.8	161.6	1.06	0.91–1.24
Bladder (C67–C68)^a^	173	183.5	224.4	0.82	0.70–0.95
Brain and other nervous system (C70–C72)^a^	110	116.7	124.2	0.94	0.77–1.13
Thyroid gland (C73)^a^	18	19.1	13.9	1.38	0.81–2.17
Hodgkin's disease (C81)^a^	29	30.8	35.1	0.88	0.59–1.26
Non-Hodgkin's disease (C82–C85 and C91.4)^a^	85	90.2	91.6	0.98	0.79–1.22
Myeloma (C90)^a^	51	54.1	52.6	1.03	0.77–1.35
Leukaemia (C91–C95, except C91.4)	127	134.7	150.9	0.89	0.74–1.06
Leukaemia excluding chronic lymphatic (C91–C95, except C91.1 and C91.4)	71	75.3	83.9	0.90	0.70–1.13
Other and unspecified	295	312.8	312.5	1.00	0.89–1.12
All cancers other than lung cancer (C00–C32 and C35–C97)	3340	3543.7	3488.2	1.02	0.98–1.05

a Exclusion of some cases for specific years, as external rates are missing (in total 46 missing cases).

O^*^, corrected for missing causes of death, O^*^=O/0.943; E=expected cases based on age and calendar year standardised national mortality rates from Eastern Germany.

**Table 3:** Relative risk for selected cancer sites by cumulative radon exposure based on internal poisson regression, 1960–2003

**Cancer site (ICD-10 code)**	**Cumulative radon exposure in working level months (WLM)**										
		**0**	**0–49**	**50–99**	**100–499**	**500–999**	**−1499**	**⩾1500**	**Total**	**ERR/100 WLM^a^**	***P*-value**
Tongue and mouth	Cases	3	12	5	11	1	6	0	38	0.045	0.50
(C01–C06)	RR^b^	1.00	1.30	3.10	3.12	0.43	6.15	—			
Pharynx	Cases	6	16	2	10	12	6	1	53	0.163	0.12
(C09–C14)	RR	1.00	0.97	0.54	1.27	2.85	3.33	1.18			
Oesophagus	Cases	19	38	7	37	15	6	3	125	−0.025	0.08
(C15)	RR	1.00	0.97	0.76	1.31	0.82	0.69	0.68			
Stomach	Cases	76	143	35	141	95	62	38	590	0.021^c^	0.04
(C16)	RR	1.00	1.21	1.42	1.34	1.16	1.51	1.77^c^			
Colon	Cases	51	74	13	66	41	44	10	299	0.017	0.26
(C17–C18)	RR	1.00	0.79	0.63	0.84	0.71	1.58	0.71			
Rectum	Cases	39	55	16	53	39	22	17	241	0.028	0.13
(C19–C21)	RR	1.00	0.77	0.97	0.91	0.89	1.03	1.58			
Liver	Cases	25	35	12	24	34	21	7	158	0.044	0.09
(C22)	RR	1.00	0.70	1.10	0.59	1.18	1.52	1.02			
Gallbladder	Cases	15	17	3	19	12	9	6	81	0.021	0.46
(C23–C24)	RR	1.00	0.70	0.57	0.85	0.70	1.05	1.37			
Pancreas	Cases	38	60	11	51	43	16	9	228	−0.001	>0.5
(C25)	RR	1.00	0.81	0.70	0.93	1.04	0.78	0.86			
Larynx	Cases	13	15	2	16	20	8	1	75	0.021	0.49
(C32)	RR	1.00	0.66	0.40	0.92	1.51	1.23	0.29			
Prostate	Cases	50	60	12	62	42	20	17	263	0.000	>0.5
(C61)	RR	1.00	0.85	0.81	0.88	0.75	0.71	1.20			
											
Kidney	Cases	26	44	12	42	23	13	11	171	0.017	0.39
(C64–C66)	RR	1.00	0.83	0.96	1.01	0.79	0.92	1.60			
Bladder	Cases	22	34	10	42	39	22	8	177	0.020	0.28
(C67–C68)	RR	1.00	1.03	1.47	1.26	1.44	1.60	1.15			
Brain and others	Cases	15	41	9	18	22	10	0	115	−0.018	0.27
(C70–C72)	RR	1.00	1.32	1.33	0.85	1.56	1.52	—			
Non-Hodgkin's disease	Cases	14	22	8	18	13	9	3	87	0.032	0.35
(C82–C85 and C91.4)	RR	1.00	0.71	1.34	1.00	1.05	1.54	1.05			
Myeloma	Cases	13	11	2	14	7	7	1	55	0.007	>0.5
(C90)	RR	1.00	0.40	0.37	0.82	0.56	1.22	0.34			
Leukaemia	Cases	22	29	5	36	17	14	4	127	0.006	>0.5
(C91–C95, excluding C91.4)	RR	1.00	0.75	0.57	1.13	0.75	1.26	0.71			
Other and unspecified	Cases	75	137	24	93	74	31	23	457	0.009	0.44
	RR	1.00	0.96	0.84	0.90	1.01	0.86	1.29			
											
All non-lung cancers	Cases	522	843	188	753	549	326	159	3340	0.014^c^	<0.001
(C00–C32, C35–C97)	RR	1.00	0.89^c^	0.94	1.00	0.99	1.20^c^	1.16			
Person-years at risk	0-year lag	256 264	781 696	102 726	290 243	191 867	92 371	47 019	1 762 208		
	5-year lag	363 845	693 782	95 229	287 756	191 037	86 643	43 913	1 762 208		
Mean cumulative radon exposure		0	11.1	71.9	263.4	732.1	1197.2	1940.8			

a ERR/WLM=excess relative risk per working level months.

b RR=relative risk.

c Statistically significant (*P*<0.05).

**Table 4:** Excess relative risk (ERR) per 100 WLM for specific cancer sites after accounting for various potential confounding factors, 1960–2003 – analyses restricted to individuals with available information on all considered confounders (*n*=56 278)

	**ERR/WLM (95% Confidence limits)**			
**Poisson regression model**	**All non-lung cancers**	**Stomach cancer**	**Larynx cancer**	**Liver cancer**
Without adjustment	0.015 (0.006; 0.023)	0.022 (0.001; 0.043)	0.020 (−0.038; 0.078)	0.042 (−0.009; 0.092)
				
*Adjusted for exposure to*				
*γ*-Radiation	0.016 (0.007; 0.026)	0.012 (−0.011; 0.035)	−0.009 (−0.067; 0.050)	0.041 (−0.015; 0.098)
LRN	0.016 (0.007; 0.026)	0.011 (−0.013; 0.034)	−0.018 (−0.077; 0.014)	0.047 (−0.009; 0.103)
Arsenic	0.014 (0.005; 0.024)	0.009 (−0.013; 0.031)	0.009 (−0.053; 0.072)	NC
Fine dust	0.011 (−0.0002; 0.023)	0.007 (−0.022; 0.036)	−0.033 (−0.129; 0.063)	0.022 (−0.045; 0.09)
Silica dust	0.012 (−0.005; 0.024)	0.012 (−0.018; 0.043)	−0.055 (−0.161; 0.050)	0.034 (−0.035; 0.101)

ERR/WLM=excess relative risk per working level months; NC=not calculable.

**Table 5:** Risk of deaths from cancers other than lung cancer combined by cumulative radon exposure in WLM in the pooled 11 miner study and this study (categorical analyses and excess relative risk per WLM)

	**11 miners' cohort study (Darby *et al*, 1995a)^a^**			**Cumulative exposure to radon in WLM**	**Present Wismut study**			
**Cumulative exposure to radon in WLM**	**Person-years**	**No. of cases**	**SMR^b^**		**Person-years**	**No. of cases**	**Relative risk**	**95% CI**
	—	—	—	0	363 845	522	1.00	
<50	295 078	405	0.98	>0–50	693 782	843	0.89	0.79–0.99
50–99	87 286	183	1.04	50–99	95 229	188	0.94	0.77–1.10
100–499	222 305	515	0.99	100–499	287 756	753	1.00	0.88–1.12
500–999	42 231	93	1.02	500–999	191 037	549	0.99	0.86–1.11
1000–1499	10 686	25	1.11	1000–1499	86 643	326	1.20	1.02–1.37
1500+	12 108	32	1.10	1500+	43 913	159	1.16	0.94–1.76
Total	669 694	1253			1 762 208	3340		
ERR/WLM^c^ (95% CI)		0.01% (−0.01 to 0.02%)				0.014% (0.006%–0.023%)		

a Risk estimates based on comparisons with external mortality rates for the time period of more than 10 years since employment.

b SMR=standardised mortality ratio, no confidence limits had been given in the original publication.

c ERR/WLM=excess relative risk per working level months.
